# Educational outcomes of Helping Babies Breathe training at a community hospital in Honduras

**DOI:** 10.1007/s40037-015-0214-8

**Published:** 2015-09-09

**Authors:** Teresa L. Seto, Meredith E. Tabangin, Srirama Josyula, Kathryn K. Taylor, Juan Carlos Vasquez, Beena D. Kamath-Rayne

**Affiliations:** 1 Division of Neonatology, Cincinnati Children’s Hospital Medical Center, Cincinnati, OH USA; 2 Division of Biostatistics and Epidemiology, Cincinnati Children’s Hospital Medical Center, Cincinnati, OH USA; 3 College of Medicine, The Ohio State University, Columbus, OH USA; 4 Harvard Medical School, Harvard University, Cambridge, MA USA; 5 Hospital Enrique Aguilar Cerrato, La Esperanza, Lindenwold, Honduras; 6 Division of Neonatology, and Global Health Center, Cincinnati Children’s Hospital Medical Center, ML 7009, 45229 Cincinnati, OH USA

**Keywords:** Neonatal resuscitation, Educational outcomes, Helping Babies Breathe

## Abstract

**Objectives:**

Helping Babies Breathe is an evidence-based curriculum designed to teach basic neonatal resuscitation in low-resource countries. The purpose of this study was to evaluate the acquisition of knowledge and skills following this training and correlation of learner characteristics to performance in a Spanish-speaking setting.

**Methods:**

Thirty-one physicians and 39 nurses completed Helping Babies Breathe training at a Honduran community hospital. Trainee knowledge and skills were evaluated before and after the training using a multiple-choice questionnaire, bag-mask ventilation skills test, and two objective structured clinical exams (OSCEs). Linear mixed-effects models were used to analyze assessment scores pre- and post-training by profession (physician or nurse) while controlling for covariates.

**Results:**

Helping Babies Breathe training resulted in significant increases in mean scores for the multiple-choice question test, bag-mask ventilation skills test, and OSCE B. Time to initiation of effective bag-mask ventilation decreased from a mean of 74.8 to 68.4 s. Despite this improvement in bag-mask ventilation, only 42 % of participants were able to initiate effective bag-mask ventilation within the Golden Minute. Although physicians scored higher on the pre-test multiple-choice questions and bag-mask ventilation, nurses demonstrated a greater mean difference in scores after training. OSCE B scores pre- and post-training increased similarly between professions. Nurses’ and physicians’ performance in simulation was not significantly different after the training. Assessment scores and course feedback indicated a need for more skills practice, particularly with bag-mask ventilation.

**Conclusions:**

When evaluated immediately after an initial workshop, Helping Babies Breathe training resulted in significant gains in neonatal resuscitation knowledge and skills. Following training, nurses, who commonly do not perform these skills in real-life situations, were able to perform at a similar level to physicians. Further studies are necessary to determine how to sustain this knowledge and skills over time, tailor the course to learner characteristics, and whether this training translates into improvements in clinical practice.

## Essentials

- Helping Babies Breathe training resulted in significant improvements in neonatal resuscitation knowledge and skills. However, scores also indicate a need to focus more on skills practice, particularly with bag-mask ventilation.
- Although physicians scored higher overall in pre-tests, nurses demonstrated a greater mean difference in scores, indicating that Helping Babies Breathe can be successful at teaching other health professionals in resuscitation skills.
- After training, nurses were able to achieve simulation performance scores similar to physicians.
- Despite improvement in bag-mask ventilation skills, participants continued to struggle to achieve ventilation by the Golden Minute, supporting the hypothesis that additional practice is needed to achieve and retain resuscitation skills.
- Further studies are necessary to determine how to maximize acquisition of and sustain Helping Babies Breathe knowledge and skills over time, tailor the course to learner characteristics, and determine whether Helping Babies Breathe translates to improvements in clinical practice.

## Background

Approximately 3 million neonatal deaths occur worldwide each year, with 98 % of these deaths occurring in low-resource countries [1]. Birth asphyxia accounts for almost a third of these neonatal deaths [2], particularly in low-resource countries, where there is a lack of skilled birth attendants proficient in neonatal resuscitation [3]. Indeed, training in neonatal resuscitation has been shown to improve clinical performance in resuscitation and reduce intrapartum-related deaths [4, 5].

Helping Babies Breathe is an evidence-based curriculum designed specifically to train birth attendants in low-resource countries in the skills of neonatal resuscitation, in order to reduce global neonatal mortality. Helping Babies Breathe focuses on the essential steps of resuscitation, including preparation for birth, evaluation of the infant, stimulation to breathe, bag-mask ventilation, and ventilation by the critical first ‘Golden Minute’® after birth [6]. Helping Babies Breathe promotes active learning using hands-on skills practice with a newborn simulator, paired learning, self-reflection, group discussion, and immediate feedback. Immediately following the training, learners are tested with four formative assessments, or systematic methods to determine learner competence and performance: a multiple-choice questionnaire, a bag-mask ventilation skills checklist, and two objective structured clinical examinations (OSCE).

Singhal et al. published a description of a formative educational evaluation of these Helping Babies Breathe assessments in Kenya and Pakistan [7]. From these study findings, modifications were made to the curriculum and assessments prior to its global launch. Subsequently, published studies in Asian and African settings have examined pre- and post-test scores and demonstrated a significant gain in resuscitation knowledge and skills immediately following Helping Babies Breathe training [7, 8]. However, these studies did not perform detailed examination of learner performance in relation to characteristics such as profession and level of experience. A greater understanding of learner performance can lead to refinement of the Helping Babies Breathe curriculum and development of the best educational methods to promote retention of neonatal resuscitation knowledge and skills that is tailored to the specific learner.

Therefore, our overall goal was to perform an in-depth analysis of the Helping Babies Breathe learner assessments to identify learner characteristics that may optimize teaching methodologies and promote the acquisition of neonatal resuscitation skills to fully achieve the goals of this training. Further, this study aims to examine the acquisition of knowledge and skills following the training in a Spanish-speaking hospital setting. Specifically, this study evaluated overall learner performance on the four Helping Babies Breathe educational assessments, assessment of trainee characteristics on performance, learner confidence, and learner feedback regarding course satisfaction. Our baseline delivery room observations of neonatal resuscitations in the hospital revealed that doctors performed most of the resuscitations and had prior training; thus, we also hypothesized that nurses may benefit more from Helping Babies Breathe than physicians, because it would give them opportunities to practise resuscitation skills with simulation, as they did not often perform these skills in real-life situations. We therefore hypothesized that all participants would have significantly improved post-test scores on all assessments when compared with pre-test scores, accounting for baseline differences in characteristics such as previous simulation training and years of medical practice. Similar scores among learners after training would illustrate the importance of providing such training to hospital staff at all levels, particularly non-physicians, who are potentially capable of performing at the same level in basic resuscitation situations.

## Methods

### Study setting

The study was exempted by the Cincinnati Children’s Hospital Medical Center Institutional Review Board and approved by the Secretary of Health, Intibucá, Honduras. Helping Babies Breathe training and assessments were performed at Hospital Enrique Aguilar Cerrato (HEAC) in La Esperanza, Honduras. HEAC serves as the delivery hospital for clinics and the rural community of western Honduras, which has a high neonatal mortality rate of 46.9 deaths per 1000 discharges [9]. There are approximately 3000 deliveries per year at this hospital.

### Participants

Study participants consisted of health care personnel at HEAC who attend deliveries or care for neonates in the hospital. In this hospital, physicians typically lead resuscitations and perform advanced neonatal resuscitation skills such as bag-mask ventilation. A total of 70 health care personnel participated in the Helping Babies Breathe training: 31 physicians (44 %) and 39 nurses (56 %). Before the course, participants completed a brief questionnaire of prior experiences that could affect test performance, including years of medical training and practice, prior neonatal resuscitation training, and prior experience with simulation. Participants were also asked to rate their confidence level in performing neonatal resuscitation and bag-mask ventilation using a Likert scale (1–5, with 5 being strongly agree).

### Course structure

The Helping Babies Breathe provider course was taught as a one-day, eight-hour-long workshop at HEAC in August 2013. Three workshops were conducted to accommodate all the staff at the hospital, with 20–25 participants per day. Each workshop consisted of both physicians and nurses, as they typically attend and perform neonatal resuscitations as a team. The course was facilitated by Helping Babies Breathe Master Trainers from the United States who were capable of speaking and teaching in Spanish. The course was taught entirely in Spanish and used the approved Spanish Helping Babies Breathe translation (‘Ayudando a Los Bebés a Respirar’). The most experienced Master Trainer led the course didactics, demonstrations, and group discussion. For small group practice and discussion, workshop participants practised in pairs and were seated in groups of six participants to one facilitator. The facilitators rotated so that participants could gain experience with each one. Before the start of the workshop, participants were oriented to the newborn simulator and then tested with three of the four learner assessments. Participants then completed the Helping Babies Breathe course using the action plan, learner workbooks, newborn simulators, and bag-mask ventilation equipment.

On the same day, immediately following the Helping Babies Breathe training, participants completed the four learner assessments, as mentioned earlier. The multiple-choice questionnaire consists of 17 multiple-choice questions that test resuscitation knowledge, and a learner must score ≥ 80 % (14 of 17 questions) to pass. The bag-mask ventilation skills checklist is a 7-item checklist of skills to perform effective ventilation and corrective measures to improve ventilation, and a learner must perform 100 % (7 of 7 steps) correctly to pass. OSCE A is a performance assessment of preparation for birth and routine newborn care, and a learner must perform ≥ 80 % (10 of 13 steps) correctly to pass, including three essential steps. Finally, OSCE B is a performance assessment of a complex resuscitation scenario that requires bag-mask ventilation, and a learner must perform ≥ 80 % (14 of 18 steps) correctly to pass, including four essential steps.

Participants were scored on their first attempts for each assessment, but were also given the opportunity to practise with the simulator afterwards until they felt comfortable with their skills and were able to pass the assessments. In order to ensure consistent scoring and decrease inter-observer variability, each rater was oriented to assessment scoring methods using a guideline developed by the study authors. In addition, each assessment was scored by a single rater, with the exception of OSCE B, which had two raters. At the end of the course, participants completed course evaluations that included confidence level, course satisfaction, and open-ended questions about the best part of the course and areas for improvement.

### Statistical analysis

Data were collected for each individual participant. Descriptive statistics (mean, standard deviation) were calculated for each assessment. Differences in characteristics between nurses and physicians were tested using chi-square tests for categorical variables or t-tests or Wilcoxon rank-sum tests for continuous variables. Performance was measured as overall scores and pass rates based on the first attempt. Pearson or Spearman’s rho correlation was used to examine relationships between assessments. To account for the hierarchical structure of the data, linear mixed-effects models were used to analyze assessment scores pre- and post-training by learner profession (physician or nurse) and an interaction effect between learner profession and time of assessment, while controlling for the covariates: years of medical practice, previous resuscitation workshop, and previous simulation experience. Separate models were built for each assessment (multiple-choice questionnaire, bag-mask ventilation, and OSCE B). We assessed models for multi-collinearity using diagnostics including variance inflation factors and condition indices. Backward elimination was used to determine the most parsimonious model controlling for important covariates for each assessment. SAS version 9.3 (SAS Institute, Cary, NC) was used to conduct the analysis. *P*-values < 0.05 were considered statistically significant.

## Results

### Characteristics of learners

When compared with nurses, physicians had greater prior resuscitation training (87 vs. 54 %, *p* < 0.01) and previous experience with simulation (90 vs. 53 %, *p* < 0.01). While physicians had a greater amount of medical training (8.0 ± 3.7 years vs. 5.1 ± 4.9 years, *p* = 0.01), nurses had more years of medical practice (10.6 ± 8.2 years vs. 5.7 ± 7.6 years, *p* = 0.01). Doctors and nurses reported attending a similar number of deliveries per month (22.7 ± 20.1 and 23.6 ± 23.4, respectively).

### Performance on educational assessments

**Fig. 1:**
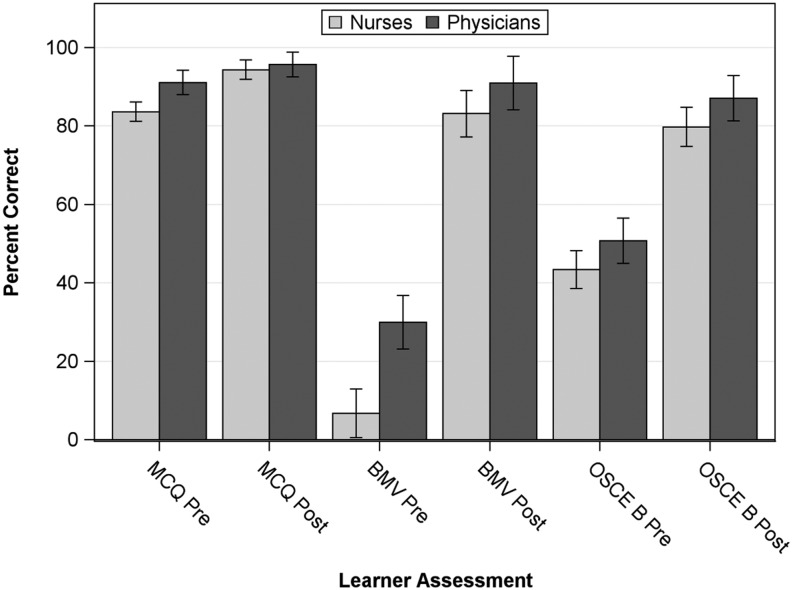
Assessment scores by profession. Assessment scores pre and post training by profession represented as percent correct of total items on each assessment. Values are the adjusted means with standard errors from linear mixed models for each assessment. Physicians scored higher than nurses on both the pre-test multiple-choice questionnaire (*MCQ*) and bag-mask ventilation skills checklist (*BVM*), though both groups significantly improved post training on all assessments

Overall, participants demonstrated statistically significant improvements in post-test scores for the multiple-choice questionnaire, bag-mask ventilation, and OSCE B (Fig. 1). Examination of the role of profession in test scores showed a significant interaction between profession and measurement occasion for the multiple-choice questionnaire, indicating that the change in scores differed between professions (Table 1). Physicians scored significantly higher than nurses on the pre-test multiple-choice questions (15.9 ± 1.1 vs. 14.2 ± 2.1). However, both physicians and nurses showed significant improvement from pre-test to post-test scores: physician post-test multiple-choice scores increased to 16.7 ± 0.7 and nurses improved to 16.0 ± 1.2. Modest increases in multiple-choice scores were associated with previous resuscitation workshop experience. Years of medical practice was included as a potential confounder; however, estimates of the regression coefficient and confidence interval suggest a weak association and may have been influenced by a low pre-test score of a participant with many years of medical practice (Table 1).

**Table 1:** Mixed effects model estimates for multiple-choice questionnaire

Parameter		95 % Confidence interval	
**Fixed effects**	**β (SE)**	**Lower**	**Upper**
Intercept	14.0 (0.29)	13.47	14.63
Profession (Physician vs Nurse)	1.27 (0.34)	0.58	1.95
Measurement occasion (Pre to post test change in score)	1.82 (0.26)	1.29	2.35
Profession by measurement occasion	− 1.04 (0.40)	− 1.85	− 0.24
Years of medical practice	− 0.03 (0.02)	− 0.06	− 0.0001
Resuscitation workshop experience (Yes vs No)	0.89 (0.26)	0.32	1.46
**Covariance**	**Estimate (SE)**	**Lower**	**Upper**
Repeated measurement within subjects	0.33 (0.21)	− 0.08	0.74
Residual	1.37 (0.23)	1.01	1.98

Bag-mask ventilation skillsscores also improved after training, though the change in scores differed by profession (Table 2). Physicians and nurses scored differently on the pre-test (2.1 ± 1.7 vs. 0.5 ± 0.9), but the scores of both groups increased post-training to 6.4 ± 1.4, and 5.8 ± 1.2, respectively.

**Table 2:** Mixed effects model estimates for bag-mask ventilation skills checklist

Parameter		95 % Confidence interval	
**Fixed effects**	**β (SE)**	**Lower**	**Upper**
Intercept	0.47 (0.22)	0.04	0.91
Profession (Physician vs Nurse)	1.63 (0.33)	0.98	2.27
Measurement occasion (Pre to post test change in score)	5.35 (0.30)	4.76	5.94
Profession by measurement occasion	− 1.08 (0.44)	− 1.96	− 0.19
**Covariance**	**Estimate (SE)**	**Lower**	**Upper**
Repeated measurement within subjects	0.10 (0.21)	− 0.32	0.52
Residual	1.64 (0.28)	1.20	2.37

OSCE B scores increased post training for both physicians and nurses. Physician scores improved from 9.5 ± 3.7 to 16.0 ± 2.5 and scores for nurses improved from 7.8 ± 3.3 to 14.3 ± 2.3 following training. Prior simulation experience was associated with increased OSCE B scores (Table 3).

**Table 3:** Mixed effects model estimates for objective structured clinical examination B (OSCE B)

Parameter		95 % Confidence interval	
**Fixed effects**	**β (SE)**	**Lower**	**Upper**
Intercept	7.27 (0.53)	6.22	8.33
Profession (Physician vs Nurse)	0.66 (0.56)	− 0.45	1.77
Measurement occasion (Pre to post test change in score)	6.70 (0.54)	5.63	7.77
Past simulation experience (Yes vs No)	1.42 (0.60)	0.21	2.62
**Covariance**	**Estimate (SE)**	**Lower**	**Upper**
Repeated measurement within subjects	− 0.50 (1.09)	− 2.64	1.64
Residual	9.19 (1.63)	6.68	13.44

Pre- and post-test pass rates for all assessments also showed marked improvements. Participants required 1.6 (range 1–3) attempts to pass bag-mask ventilation skills test, 1.1 (range 1–2) attempts to pass OSCE A, and 1.4 (range 1–4) attempts to pass OSCE B. The ‘Golden Minute’® is a term coined by Helping Babies Breathe to denote the critical first minute after birth during which neonates should begin breathing spontaneously or receive assistance with adequate and effective bag-mask ventilation. Achievement of ventilation by the Golden Minute improved from 27.9 % pre-test to 42.4 % post-test (*p* = 0.02). The time to initiation of bag-mask ventilation decreased from 74.8 ± 41.1 s to 68.4 ± 25.9 s following training (*p* = 0.12).

### Confidence level

Confidence in neonatal resuscitation increased from 4.01 pre-training to 4.59 after training, with no significant difference seen between physicians and nurses. Similarly, confidence in bag-mask ventilation increased from 4.11 pre-training to 4.67 after training. Confidence level did not correlate to pre- or post-test performance.

### Qualitative feedback, course satisfaction

Immediately following the Helping Babies Breathe course, participants completed a course evaluation that utilized a Likert scale scoring system (scores 1–5, with 1 = strongly disagree and 5 = strongly agree). Survey responses were positive, with the highest mean scores seen for items pertaining to satisfaction with the course, simulators, flipchart, and group discussions, and opportunities to practise bag-mask ventilation (mean scores 4.83–4.98). Lower mean scores were seen for items related to time to learn and practise the Helping Babies Breathe curriculum (mean scores 4.41–4.49).

Qualitative feedback was also obtained from open-ended questions. When asked about the best part of the course, the dominant response was the ability to practise skills. Further, participants liked practising with the simulator, particularly with a focus on bag-mask ventilation. In response to how the course could be improved, the predominant response was a request for more time, with several suggesting a two-day workshop in order to practise skills. Additional comments focused on a desire for real-life demonstrations, videos, and advanced resuscitation skills.

## Discussion

Since its release in 2009, the use of the Helping Babies Breathe curriculum has helped frontline health care workers to improve their resuscitation skills in simulated practice and impact neonatal mortality and stillbirth rates in low-resource settings [8, 10–13]. To our knowledge, this is the first published study of Helping Babies Breathe conducted in a Latin-American country and using the approved Spanish translation. Helping Babies Breathe training at this Spanish-speaking study site resulted in improved neonatal resuscitation knowledge and skills in the immediate post-workshop period, as demonstrated by statistically significant increases in mean test scores and pass rates after training. These findings are similar to those seen at other Helping Babies Breathe study sites [7, 8, 10, 12]. However, compared with other studies done in Africa and Asia, our study evaluated pre- and post-training performance in both knowledge (multiple-choice questionnaire) and skills (bag-mask ventilation and OSCEs).

Despite significant improvements in overall test scores and pass rates following training, scores and improvement were disproportionate for those assessments that involved demonstration of skills (bag-mask ventilation, OSCE A, OSCE B) versus demonstration of knowledge (multiple-choice questionnaire), a phenomena that has been seen in other resuscitation trainings as well [14, 15]. As described by George Miller’s pyramid of clinical competence in medical education, acquisition of knowledge occurs earlier and more easily than acquisition of skills [16]. Miller’s framework of progressively more complex and higher-level thinking moves from knowledge (‘knows’, multiple-choice questionnaire), to demonstration of skills (‘knows how’, bag-mask ventilation), to performance assessments (‘shows how’, OSCE). In our study, participants demonstrated mastery of neonatal resuscitation knowledge, or the ‘knows’ level of Miller’s pyramid, as evidenced by high post-test scores and pass rates for the multiple-choice questionnaire. Participants struggled more with assessments that required skills and higher-level performance, as evidenced by lower pass rates and scores compared with the multiple-choice questionnaire. Furthermore, we found only mild correlation between knowledge assessments (multiple-choice questionnaire) and skills assessments including the bag-mask ventilation and OSCE B), indicating that more focused learning and feedback are likely required for the mastery of skills rather than knowledge performance, as hypothesized by Singhal et al. [7]. Qualitative survey feedback also indicated a desire for more skills practice.

Examination of bag-mask ventilation performance, a key skill in Helping Babies Breathe training, revealed dramatic improvements in post-test scores, indicating that Helping Babies Breathe is an effective method of teaching this skill. Interestingly, participants rated high confidence levels with regards to bag-mask ventilation prior to the Helping Babies Breathe training, despite low pre-test scores. Scores for the bag-mask ventilation checklist, as well as bag-mask ventilation-related steps in OSCE B, showed significant improvement from pre- to post-test. Despite improvement in bag-mask ventilation skills, participants continued to struggle to achieve ventilation by the Golden Minute, with only 42.4 % achieving that goal following training. Feedback from the post-course survey supported the need for more time to practise bag-mask ventilation skills. Similarly, Singhal et al. found that following Helping Babies Breathe training, participants continued to demonstrate difficulty with bag-mask ventilation, citing that this is a complicated skill that may require more time to master than allowed for in the time allotted for Helping Babies Breathe training [7].

While our study demonstrated similar improvements in pre- and post-Helping Babies Breathe training performance on learner assessments, we further examined the effect of learner characteristics on performance. The model for the assessment of resuscitation knowledge indicated those with previous resuscitation workshop experience had higher multiple-choice scores. In the assessment of a complex resuscitation scenario (OSCE B), the model indicated those with past simulation experience had higher OSCE B scores. In their educational evaluation of Helping Babies Breathe, Singhal et al. had demonstrated differences between individuals who were trained to be Helping Babies Breathe facilitators and learners [7]. The study described that facilitators were chosen based on their experience in labour/delivery and neonatology, and it was not further specified if they had a specific profession. Singhal et al. noted that both facilitators and learners improved in both the multiple-choice questions and bag-mask ventilation assessments; however, neither facilitators nor learners were able to pass the bag-mask ventilation assessment on a single attempt [7].

We examined learner differences based on profession and adjusted for other differences in baseline characteristics, such as years of medical practice, resuscitation workshop experience, and simulation experience. Physicians scored higher than nurses, as expected, indicating a greater knowledge and skills base prior to Helping Babies Breathe. Nurses did not score well on bag-mask ventilation prior to Helping Babies Breathe training, as this is a skill with which they likely did not have previous experience. Interestingly, however, physicians also performed poorly on the pre-test bag-mask ventilation skills checklist, despite likely having learned these skills in medical training and as the primary providers of bag-mask ventilation and resuscitative steps at this study hospital. Following Helping Babies Breathe training, there was a smaller gap in scores between physicians and nurses for the multiple-choice questionnaire and bag-mask ventilation. This mirrors findings by Hoban et al. who examined differences in doctors vs. non-doctors before and after training in an abridged version of the multiple-choice questionnaire only, and found that the training eliminated differences in multiple-choice questionnaire scores [11].

Despite the difference in prior education and professional training seen in our study, Helping Babies Breathe was beneficial and appropriate regardless of profession, as demonstrated by statistically significant gains in post-test scores for both physicians and nurses. It is important to note that nurses were able to achieve scores similar to physicians after training and simulated performance, despite lack of previous training in neonatal resuscitation and fewer opportunities to perform basic resuscitation skills in real-life delivery situations. This study shows that Helping Babies Breathe can be an effective method to teach bag-mask ventilation to nurses, who are often the first responders to neonatal resuscitations. However, nurses did have a lower pass rate on the bag-mask ventilation and OSCE B post-training assessments, indicating that the skill of bag-mask ventilation is likely one with which they will require further practice after initial training. Therefore, this study also points to the potential need to tailor training and subsequent refreshers for different levels of health workers and settings. An assessment of learner background and experience should be performed prior to the start of Helping Babies Breathe training, and training should then take into account learner characteristics such as profession and prior bag-mask ventilation experience when allocating time for knowledge and skills practice.

There were several limitations for this study. First, Helping Babies Breathe may have been a participant’s first encounter with bag-mask ventilation equipment, particularly for the nurses. Thus, the learner may require more time to become proficient with the complexity and skills involved with bag-mask ventilation, and this unfamiliarity may contribute to low test scores [7]. Similarly, the use of a simulator may be an unfamiliar concept for course participants and may adversely affect test scores, although the majority of participants (66 %) had previous simulation experience and we adjusted for this covariate in the model for OSCE B assessments. Further, Helping Babies Breathe may be limited in effectiveness by the time constraints of the course and assessments, as they were conducted over a single day in order to accommodate the schedules of the hospital staff and instructors. Learners were assessed immediately prior to and after completing the Helping Babies Breathe course, but it is likely that more time is needed to consolidate knowledge and practise neonatal resuscitation skills, as suggested by survey feedback, and similar to what was seen by Singhal et al. in their educational evaluation in Kenya and Pakistan [7].

## Conclusions

Helping Babies Breathe has had a significant clinical impact on the reduction of neonatal mortality and has been successfully implemented in over 70 countries [8, 13, 17]. Now that the clinical effectiveness of Helping Babies Breathe has been shown, closer examination of the educational aspects of Helping Babies Breathe, particularly acquisition of skills, are necessary to maximize the future impact of this course. Results from this study demonstrate that while knowledge can be easily acquired, it is more difficult to learn neonatal resuscitation skills, achieve competence, and translate skills to real-life clinical scenarios [12]. Further, this study identified specific aspects of Helping Babies Breathe that are more challenging to master, particularly bag-mask ventilation. Awareness of the difficulty of acquisition of skills should lead to an increased focus on the best instructional methods to teach skills such as bag-mask ventilation in order to achieve effective ventilation by the Golden Minute, taking into consideration that participants in courses will have different training and clinical backgrounds.

Furthermore, it is well known that resuscitation skills deteriorate rapidly over time [15, 18–20]. While this phenomenon is generalizable to neonatal resuscitation courses overall, several Helping Babies Breathe follow-up studies have raised concern for the potential decline in basic resuscitation skills over time [8, 10]. Further studies are necessary to determine the retention of knowledge and skills following Helping Babies Breathe training, as well as the frequency of training and skills practice necessary to maintain skills, particularly when participants will have different rates of utilizing acquired skills in real life delivery situations. Correlations of Helping Babies Breathe training to clinical performance and outcomes are also necessary. Further investigation is needed of the process by which a learner masters skills such as bag-mask ventilation so that these skills can be successfully performed repeatedly in a clinical setting, adherence to the Helping Babies Breathe action plan, and measures of stillbirth and neonatal mortality rates over time. Together, these further studies can lead to development of the best instructional and assessment methods so that Helping Babies Breathe can maximize acquisition of neonatal knowledge and skills that will lead to sustained reductions in global neonatal mortality.
